# Early Chronotype and Tissue-Specific Alterations of Circadian Clock Function in Spontaneously Hypertensive Rats

**DOI:** 10.1371/journal.pone.0046951

**Published:** 2012-10-02

**Authors:** Martin Sládek, Lenka Polidarová, Marta Nováková, Daniela Parkanová, Alena Sumová

**Affiliations:** Department of Neurohumoral Regulations, Institute of Physiology Academy of Sciences of the Czech Republic v.v.i., Prague, Czech Republic; Karlsruhe Institute of Technology, Germany

## Abstract

Malfunction of the circadian timing system may result in cardiovascular and metabolic diseases, and conversely, these diseases can impair the circadian system. The aim of this study was to reveal whether the functional state of the circadian system of spontaneously hypertensive rats (SHR) differs from that of control Wistar rat. This study is the first to analyze the function of the circadian system of SHR in its complexity, i.e., of the central clock in the suprachiasmatic nuclei (SCN) as well as of the peripheral clocks. The functional properties of the SCN clock were estimated by behavioral output rhythm in locomotor activity and daily profiles of clock gene expression in the SCN determined by *in situ* hybridization. The function of the peripheral clocks was assessed by daily profiles of clock gene expression in the liver and colon by RT-PCR and *in vitro* using real time recording of *Bmal1-dLuc* reporter. The potential impact of the SHR phenotype on circadian control of the metabolic pathways was estimated by daily profiles of metabolism-relevant gene expression in the liver and colon. The results revealed that SHR exhibited an early chronotype, because the central SCN clock was phase advanced relative to light/dark cycle and the SCN driven output rhythm ran faster compared to Wistar rats. Moreover, the output rhythm was dampened. The SHR peripheral clock reacted to the dampened SCN output with tissue-specific consequences. In the colon of SHR the clock function was severely altered, whereas the differences are only marginal in the liver. These changes may likely result in a mutual desynchrony of circadian oscillators within the circadian system of SHR, thereby potentially contributing to metabolic pathology of the strain. The SHR may thus serve as a valuable model of human circadian disorders originating in poor synchrony of the circadian system with external light/dark regime.

## Introduction

Spontaneously hypertensive rats (SHR) have been widely recognized as an animal model for various diseases, including essential hypertension [1] and metabolic syndrome [2]. Thus, this model can be used for studies on interference of these diseases with other regulatory systems. The cardiovascular and metabolic functions are both under temporal control of the endogenous timekeeping system. The role of the system is to optimize these and other physiological functions in anticipation of daily changes in the external environment. To fulfill this role, the system drives rhythms in physiological functions with an about-a-day, i.e., circadian, period. The rhythms are regularly entrained by external cues and run in accordance with the solar day. In mammals, the system consists of central clock located in the suprachiasmatic nuclei (SCN) in the hypothalamus and of numeral peripheral clocks in various tissues and cells in the body, including the heart, liver, kidney, lung, intestine etc. (for review, see [3]). The circadian oscillations are generated at the cellular level by a molecular mechanism that consists of transcriptional-translational feedback loops, which are formed by several clock genes and their protein products [4]. The transcriptional activator, a dimer of the clock proteins CLOCK and BMAL1, binds to E-box elements in the promoter regions of clock genes *Per1*, *Per2*, *Cry1*, *Cry2*, *Rev-erbα* and *Rora*, driving their rhythmic expression [5], [6], [7], [8]. PER and CRY proteins accumulate in the cytoplasm, form complexes and translocate into the nucleus where they inhibit the CLOCK/BMAL1-mediated transcription and produce rhythms in the expression of these clock genes [9], [10], [11]. Furthermore, REV-ERBα and RORa also enter the nucleus, where they repress or activate *Bmal1* transcription, respectively. Thus, the rhythm of *Bmal1* expression is in anti-phase to the rhythm of *Per* and *Cry* [12], [13]. The accurate fine-tuning of speed of the clock is provided by posttranslational modifications (for review, see [14]). Importantly, the clock mechanism drives rhythmically expression of other, so called clock-controlled, genes coding transcription factors or functional proteins involved in various physiological pathways (for review, see [15], [16]). To ensure proper temporal coordination of bodily functions with external daytime, the endogenous circadian oscillations are regularly synchronized by light/dark cycle, via the retinorecipient part of the SCN (for review, see [17]). The entrained SCN clock then relays the external time information to the peripheral clocks in the body. The peripheral clocks can also be entrained independently of the SCN signaling, which can occur with a regular feeding regime or external temperature cycle (for review, see [3]).

While deficiencies in circadian timing can result in cardiovascular and metabolic diseases [18], [19], [20], these diseases can also cause impairment of the circadian system [21], [22], [23] (for review, see [24]). Several lines of evidence suggested that in SHR the temporal control of physiology may be affected, namely because of the dampened amplitudes of day/night rhythms in blood pressure [25] and the aberrant sleeping patterns [26]. The findings regarding the genetic abnormalities of SHR supported this hypothesis. In the SCN, elevated expression of *vasoactive intestinal peptide (VIP)* gene was found in SHR compared with Wistar Kyoto (WKY) rats [27]. VIP plays a major role in the communication among individually oscillating SCN cells, and its presence seems to be conditional for a high amplitude SCN circadian rhythmicity [28]. At the level of the peripheral clocks, tissue-specific differences in clock-related gene expression were also found in SHR. Higher amplitude and overall expression of clock genes *Per2*, *Bmal1*, *Clock*, and clock-controlled genes *Dbp* were reported in the heart, but not in the aorta, of SHR as compared with WKY rats [29]. Finally, Woon and colleagues recently suggested a potential direct link between the SHR pathological phenotype and the circadian system when they identified polymorphisms in the SHR *Bmal1* promoter that were associated with metabolic syndrome [30]. The polymorphisms might play an important role in the SHR phenotype because *Bmal1* promoter functions as one of the main “hubs” connecting the circadian system with metabolism (for review, see [31]). Surprisingly, studies investigating the relationship between the circadian and metabolic systems in SHR are rather sparse. Also, a detailed analysis of the SHR circadian clock at the molecular level is still lacking. Therefore, the aim of the present study was to characterize the circadian system of SHR and compare it to that of Wistar rats, which is a well-described and widely used animal model of a normotensive rat strain without metabolic and cardiac pathology. In this study, the period, amplitude and phase of the central clock in SHR was compared to control rats as measured by behavioral output rhythm in locomotor activity and daily profiles of clock gene expression in the SCN. To study the interaction of the circadian and metabolic systems, the phases and amplitudes of oscillations of the peripheral clocks related with the metabolic system in SHR were compared with those of control rats by examining the daily profiles of clock gene expression in the liver and colon. The period of SHR peripheral clock was compared to that of controls *in vitro* using real-time recordings of the *Bmal1-dLuc* reporter. To elucidate a potential impairment in circadian control of the metabolic pathways in SHR, daily profiles of metabolism-relevant gene expression in the liver and colon of SHR were compared with those in control rats. The results revealed unexpected differences in the organization of the circadian system in SHR and Wistar rats, which might potentially contribute to the metabolic phenotype of the rat strain.

## Methods

### Experimental Animals

Two-month-old male SHR/Ola (Institute of Physiology, Academy of Sciences of the Czech Republic) and Wistar:Han rats (Velaz s.r.o., Czech Republic) were maintained at a temperature of 21±2°C in an alternating light – dark (LD) regime with 12 h of light and 12 h of darkness per day. The lights were turned on at 06:00 h and off at 18:00 h. Light was provided by overhead 40-W fluorescent tubes, and illumination was between 50 and 300 lux, depending on the cage position in the animal room. The animals had free access to food and water throughout the experiment.

All experiments were approved by Animal Care and Use Committee of the Institute of Physiology in agreement with Animal Protection Law of the Czech Republic as well as European Community Council directives 86/609/EEC. All efforts were made to ameliorate the suffering of animals.

### Experimental Protocol

For behavioral study, rats of both strains were maintained under LD12:12 for 4 weeks and then released into constant darkness (DD) for two or more weeks. The light was not turned on at the usual time of dark-to-light transition, which was designated as circadian time 0 (CT0). During the entire experiment, locomotor activity was monitored.

For gene expression study, rats of both strains were maintained under LD12:12 for four weeks. On the day of sampling, the animals were released into DD. To determine a daily profiles in gene expression, animals were sampled during the first day in DD, starting at CT0, every 4 h throughout the whole 24-h circadian cycle. Three to five rats per each time point were killed by decapitation under deep anaesthesia (i.p. injection of thiopental, 50 mg per kg) and samples of the brain, liver and colon were collected.

### Locomotor Activity Monitoring

SHR and Wistar rats were maintained individually in cages equipped with infrared movement detectors attached above the center of the cage top, which enabled detection of locomotor activity across the whole cage. A circadian activity monitoring system (Dr. H.M. Cooper, INSERM, France) was used to measure activity every minute and double-plotted actograms were generated to visualize the data. The resulting data, including calculations of the chi-square periodograms with *P*<0.001, activity and activity/rest ratio were analyzed using ClockLab toolbox (Actimetrics, Illinois, USA). At least three independent experiments were performed for the determination of endogenous periods and activity/rest ratios. In each experiment rats of both strains were monitored simultaneously. The free-running period under DD was calculated for each animal. Thereafter, the actograms were re-plotted using the calculated individual periods and subsequently used to calculate total activity and activity/rest ratios during DD. For analysis of behavior under LD, the actograms were plotted with 24 h period.

### Tissue Sampling

Brains were removed, immediately frozen on dry ice and kept at −80°C. They were sectioned into five series of 12 µm thick slices in an alternating order throughout the whole rostro-caudal extent of the SCN. The sections were processed for *in situ* hybridization in order to determine gene expression profiles in the SCN.

Samples from the liver were dissected and immersed into RNAlater stabilization reagent (Qiagen, Valencia, USA). Dissected samples of the distal part of the colon, just above the pelvic brim, were rinsed with phosphate-buffered saline and cut longitudinally. The mucosal layer was gently scrapped, which yielded material rich in epithelial cells that was next immersed in RNAlater stabilization reagent (Qiagen, Valencia, USA). All samples were stored at 4°C for no longer than 1 week prior to isolation of total RNA and subsequent real-time RT-PCR.

For protein analysis, colon samples were collected during the day (at 10:00 h). The colon was dissected, rinsed with cold saline (150 mM NaCl) and cut longitudinally. Sections were frozen on dry ice and stored at −20°C until processed for immunohistochemistry.

For preparation of primary rat fibroblasts, samples of skeletal muscle were collected, dissected and processed as described below.

### In situ Hybridization

The cDNA fragments of rat *rPer1* (980 bp; corresponds to nucleotides 581–1561 of the sequence in GenBank with accession no. AB002108), *Per2* (1512 bp; 369–1881; GenBank NM_031678), *rRev-erbα* (1109 bp; 558–1666; Genbank BC062047) and *rBmal1* (841 bp; 257–1098; GenBank AB012600) were used as templates for *in vitro* transcription of cRNA probes. Probes were labeled using ^35^S-UTP, and the *in situ* hybridizations were performed as previously described [32]. The brain sections were hybridized for 20 h at 60°C. Following a post-hybridization wash, the sections were dehydrated in ethanol and dried. Finally, the slides were exposed to BIOMAX MR film (Kodak, USA) for 10–14 days and developed using the ADEFO-MIX-S developer and ADEFOFIX fixer (ADEFO-CHEMIE Gmbh, Germany). Brain sections from all experimental groups were processed simultaneously under identical conditions. Autoradiographs of sections were analyzed using an image analysis system (Image Pro, Olympus, New York, USA) to detect relative optical density (OD) of the specific hybridization signal.

### RNA Isolation and Real-time RT-PCR

Total RNA was extracted from the liver by rotor disruption and from the colon by sonication and subsequently purified using RNeasy Mini kit (Qiagen, Valencia, USA) according to the manufacturer’s instructions. RNA concentrations were determined by spectrophotometry at 260 nm, and RNA quality was assessed by electrophoresis on a 1.5% agarose gel. Moreover, the integrity of randomly selected samples of total RNA was tested using an Agilent 2100 Bioanalyzer (Agilent Technologies, Santa Clara, USA).

The RT-PCR method used to detect the clock genes was described previously [33]. Briefly, 1 µg of total RNA was reverse transcribed using the SuperScript VILO cDNA synthesis kit (Invitrogen, Carlsbad, USA) and random primers. Diluted cDNA was then amplified on LightCycler480 (Roche, Basel, Switzerland) using the Express SYBR GreenER qPCR SuperMix (Invitrogen, Carlsbad, USA) and corresponding primers (for primer sequences see Table S1). Relative quantification was achieved by using a standard curve and subsequently normalizing the gene expression to *β2-microglobulin* (B2M). *β2-microglobulin* has been used as a housekeeping gene previously [33], [34]. Its expression was stable throughout the day and did not vary between the analyzed tissues.

### Immunohistochemistry

To compare the spatial distribution of BMAL1-immunoreactive cells in the colon of SHR and controls, 12-µm-thick coronal sections of colon were cut, mounted on slides, fixed in 4% paraformaldehyde in PBS and processed for immunohistochemistry using the standard avidin-biotin method with diaminobenzidine as the chromogen (Vector Laboratories, Peterborough, UK) as described elsewhere [34] The BMAL1 antibody was raised against the C-terminal 15 residues of mBMAL1 (GLGGPVDFSDLPWPL) using the Sigma-Aldrich custom peptide antibody service and was characterized previously [35]. As controls for background staining, parallel sections were treated simultaneously through the immunohistochemical procedure without incubation with the specific primary antibody. Colonic sections from 3 Wistar rats and 3 SHR were examined.

### Cell Culture and Real-time Bioluminescence Monitoring

Primary rat fibroblasts were prepared from adult Wistar and SHR rat skeletal muscle. Minced fragments were incubated 40 minutes in 0.5 mg/ml collagenase (Sigma-Aldrich, St. Louis, USA) in DMEM (D6546, Sigma) with penicillin-streptomycin (1/100, Sigma) and gentamycin (1 µg/ml, Sigma). Digestion was stopped with fetal calf serum (FCS) (Sigma). The suspension was filtered through 45 µm sifter, washed with medium and plated in fresh DMEM containing 20% FCS, 1/100 Glutamax-I CTS (Gibco, Carlsbad, USA) and antibiotics. Fibroblasts emigrating from tissue pieces were allowed to reach 80% confluence, trypsinized and then grown in DMEM (10% FCS, 1/100 Glutamax, antibiotics) until spontaneously immortalized cells emerged. These cells were used for experiments between passages 5–12. Fibroblasts were plated in 35 mm dishes in DMEM without antibiotics and transfected after reaching 70–80% confluence on the next day by 5 µg Bmal1-dLuc (*Bmal1* promoter in destabilized luciferase-containing plasmid [36], kind gift of M. H. Hastings, MRC-LMB, UK) and 15 µl Genejuice (Merck, Darmstadt, Germany). The medium was changed the next day and cells were kept in humidified CO_2_ incubator for 10–15 days. The medium was then changed with fresh one containing antibiotics every 4–5 days. On the day of the experiment, the cells were synchronized with 10 µM Forskolin (Sigma) or 50% horse serum (Sigma) for 1 h or left untreated. The medium was then replaced with recording medium that contained 8.3 g/l DMEM without phenol red (D5030, Sigma), 4.5 g/l glucose, 1/100 penicillin-streptomycin, 1 µg/ml gentamycin, 0.35 g/l NaHCO_3_, 10 mM HEPES, 1/100 Glutamax, 10% FCS and 0.1 mM luciferin-EF (Promega, Madison, USA). Dishes were sealed with glass cover slips and vacuum grease and then placed into the Lumicycle (Actimetrics, Illinois, USA) for luminescence recording. Both Wistar and SHR fibroblasts were transfected and recorded simultaneously. Transfections and recordings were repeated and assessed in 2- to 3- independent experiments for each rat strain and condition.

### Statistical Analysis

The differences in locomotor activity (i.e., the values of total activity, activity/rest ratio, period, etc.) between SHR and Wistar rats were evaluated by Student’s *t* test with *P*<0.05 required for significance.

For gene expression profiles, data were fitted with single cosine curves, defined by the equation Y = mesor+(amplitude*cos(2*π*(X-acrophase)/wavelength)) with a constant wavelength of 24 h. The amplitude (i.e., difference between the peak or trough and the mean value of a cosine curve), acrophase (i.e., phase angle of the peak of a cosine curve), mesor (i.e., average value around which the variable oscillates) and coefficient of determination R^2^ (i.e., goodness of fit) of the rhythms were compared. The least-squares regression method implemented in Prism 5 software (GraphPad, La Jolla, USA) was applied. The profiles with levels of R^2^ higher than 0.2 were considered to fit the cosine curve and exhibit a circadian rhythm. The differences in acrophases and mesors between the profiles in SHR and control rats were evaluated by Student’s *t* test with *P*<0.05 required for significance.

For quantitative analysis of bioluminescence data software package supplied with Lumicycle (Actimetrics) was used. For period calculations, the raw data were baseline-corrected by 24-h running average. Start of recording in Lumicycle (i.e., time of recording medium application) was set as time 0 h. Time frame 24–144 h was analyzed by fitting a damped sin curve. The resulting period was an average ± SD of 30–48 individual dishes of each experimental group. The differences between the periods of SHR and control rats were evaluated by Student’s *t* test with *P*<0.05 required for significance.

## Results

### Circadian Control of Locomotor Activity in SHR Differs from Wistar Rats

**Figure 1:**
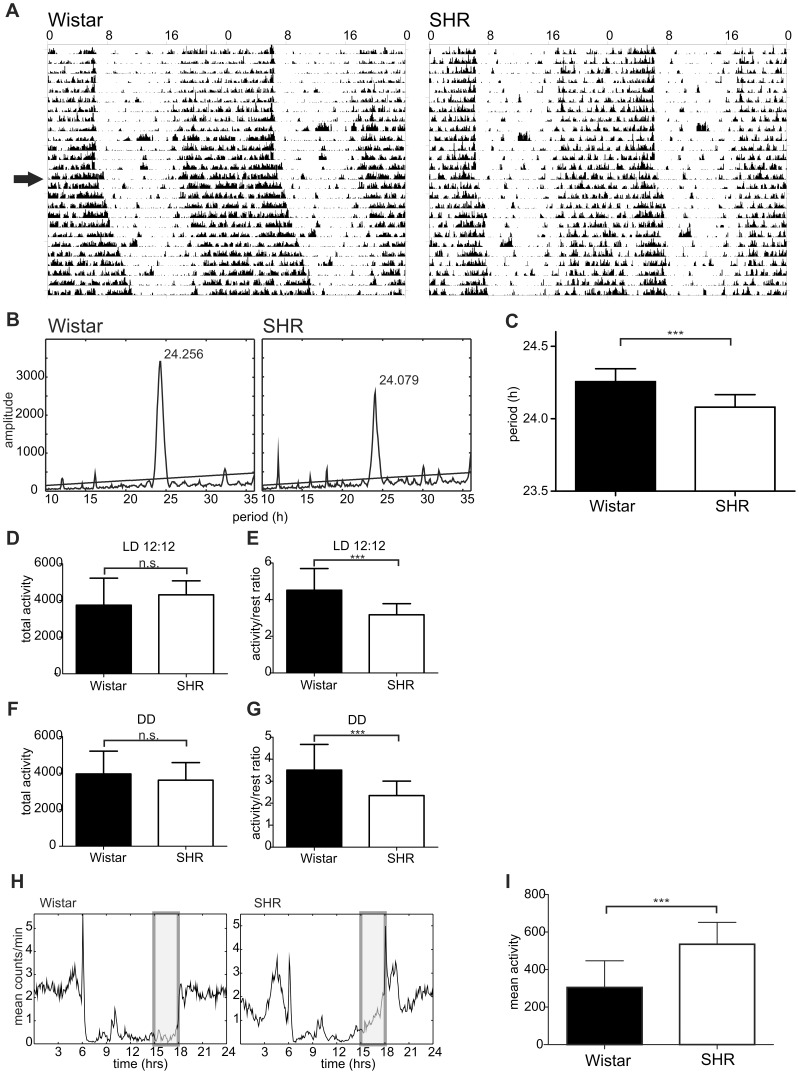
Behavioral activity of Wistar rat and SHR. **A)** Representative double-plotted actograms of the locomotor activity in a Wistar rat (left) and SHR (right) maintained under a light/dark regime with 12 h of light and 12 h of darkness (LD12:12) and released into constant darkness (DD) (arrow); **B)** Cumulative periodogram of 14 Wistar rats and 28 SHR maintained under DD for 2 weeks; **C)** Comparison of free-running periods between the Wistar rats and SHR. **D)** Total activity during the 24 h in Wistar rats and SHR maintained under LD12:12; **E)** Activity/rest ratio during the 24 h in Wistar rats and SHR maintained under LD12:12; **F)** Total activity during the circadian cycle in Wistar rats and SHR maintained under DD; **G)** Activity/rest ratio during the circadian cycle in Wistar rats and SHR maintained under DD; **H)** Cumulative daily locomotor activity profiles of Wistar rats (left) and SHR (right) maintained under LD12:12 for 17 days. Shaded rectangles represent intervals of 3 h before the light-to-dark transition (15:00–18:00 h) when activity was compared in I; **I)** Comparison of activity level in Wistar rats and SHR during the 3 h interval before the lights were turned off (shaded area in H). For details, see Methods. *** (P<0.001), n.s. (non-significant).

Representative actograms of SHR and Wistar rats maintained under LD12:12 and released into DD are shown in Fig. 1A. Period analysis revealed that the free-running period was 24.08±0.02 h (n = 28) and 24.26±0.02 h (n = 14) for SHR and Wistar rats, respectively (Fig. 1B). The free-running period of SHR was significantly shorter than that of the Wistar rats (t = 6.180, DF = 40, P<0.001) (Fig. 1C).

Under LD12:12, total locomotor activity during 24 h was not different between rat strains (t = 1.028, DF = 43, P = 0.310) (Fig. 1D). However, the activity/rest ratio (i.e., a ratio of the activity measured during the interval from ZT12 to ZT0 and from ZT0 to ZT12) was significantly lower in SHR compared to Wistar rats (3.17±0.09 vs. 4.52±0.32, respectively; t = 5.566, DF = 56, P<0.001) (Fig. 1E). Under DD, total activity per circadian cycle in SHR was also not different from that of Wistar rats (t = 1.028, DF = 43, P = 0.310) (Fig. 1F), but the subjective night/subjective day activity/rest ratio was again significantly lower in SHR compared to that of Wistar rats (2.35±0.11 vs. 3.51±0.26, respectively; t = 4.895, DF = 57, P<0.001) (Fig. 1G). A detailed analysis of the increased daytime activity in SHR revealed that under LD cycle, SHR were significantly more active, especially shortly before the lights were turned off (Fig. 1H). The relative activity measured during a 3 h interval before the lights-off (15:00–18:00 h) in Wistar rats (305±45, mean ± S.E.M., n = 10) was significantly lower than that in SHR (535±29, mean ± S.E.M., n = 16) (t = 4.503, P<0.001) (Fig. 1I).

Therefore, in SHR, the overall activity did not differ but their circadian rhythm in locomotor activity, under both LD and DD conditions, exhibited significantly lower amplitude compared to Wistar rats. Whereas under LD conditions control rats became active at the time of the lights-off, the SHR activity increased already before the light offset.

### Clock in the SCN is Phase-advanced in SHR Compared with Wistar Rats

**Figure 2:**
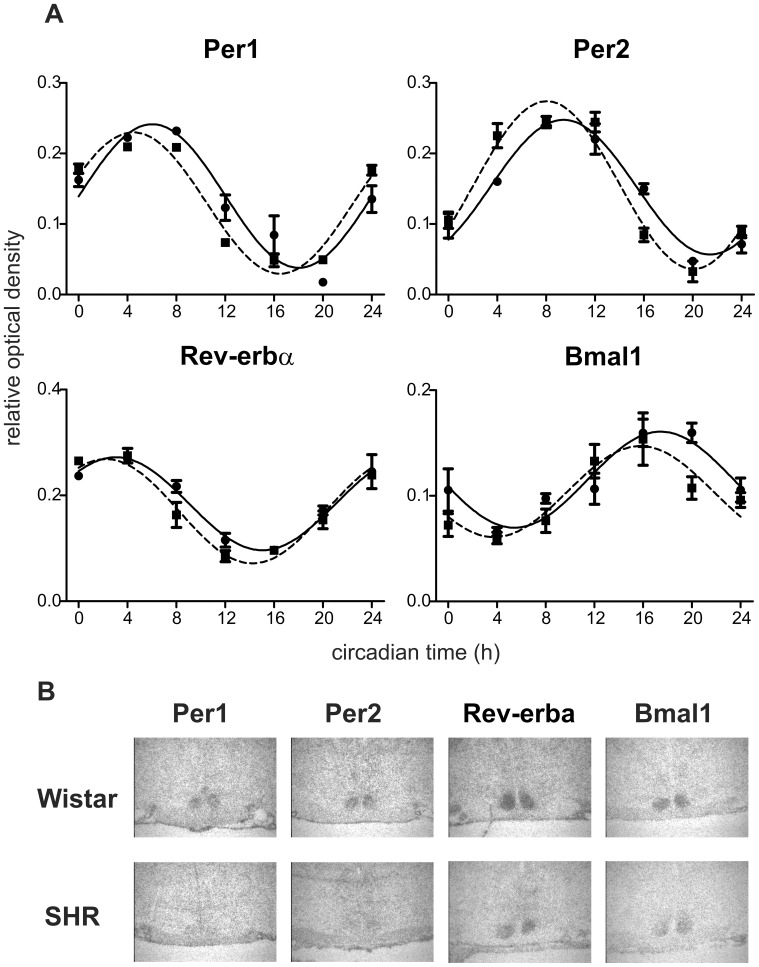
Clock gene expression in the suprachiasmatic nucleus of Wistar rats and SHR. **A)** Clock gene expression profiles in the SCN. Wistar rats (full line) and SHR (dashed line) were maintained under LD12:12, released into darkness and sampled in 4-h intervals during the 24 h. Daily profiles of *Per1*, *Per2*, *Rev-erbα* and *Bmal1* mRNA levels were determined by *in situ* hybridization. Data are expressed as relative optical density measured in the middle section of the SCN. Time is expressed as circadian time (h) with CT0 corresponding to lights-on in the previous LD cycle (for details, see Methods). Data were fitted by cosine curves and each point represents the mean ± S.E.M. of 3 (SHR) or 5 (Wistar rat) animals. **B)** Representative autoradiographs of the SCN sections in Wistar rats and SHR examined by *in situ* hybridization for *Per1*, *Per2*, *Rev-erbα* and *Bmal1* expression at CT12, 16, 8 and 20, respectively (magnified 48×).

Daily profiles of *Per1*, *Per2*, *Rev-erbα* and *Bmal1* mRNA levels (Fig. 2) were determined by *in situ* hybridization in the SCN of SHR and Wistar rats maintained under LD12:12 and sampled on the first day in constant darkness. A cosinor analysis revealed that all of the profiles exhibited circadian rhythms because R^2^ values of the cosine fits were high (Table S2). The acrophases of all studied clock gene expression profiles in SHR (Table S2) were significantly earlier compared with controls (*Per1*: t = 7.734, DF = 6, P<0.001; *Per2*: t = 7.059, DF = 6, P<0.001; *Rev-erbα*: t = 3.688, DF = 6, P = 0.010); *Bmal1*: t = 3.810, DF = 6, P = 0.009). Therefore, *Per1*, *Per2* and *Rev-erbα* and *Bmal1* expression profiles in the SCN of SHR were advanced relative to those of the controls. Neither the amplitudes nor the mesors of the profiles (Table S2) in SHR did significantly differ from those in controls. Therefore, the clock gene expression rhythms in the SCN of SHR were significantly advanced, but not suppressed, compared to those of controls.

### Clock in the Colon but not in the Liver is Altered in SHR Compared to Wistar Rats

**Figure 3:**
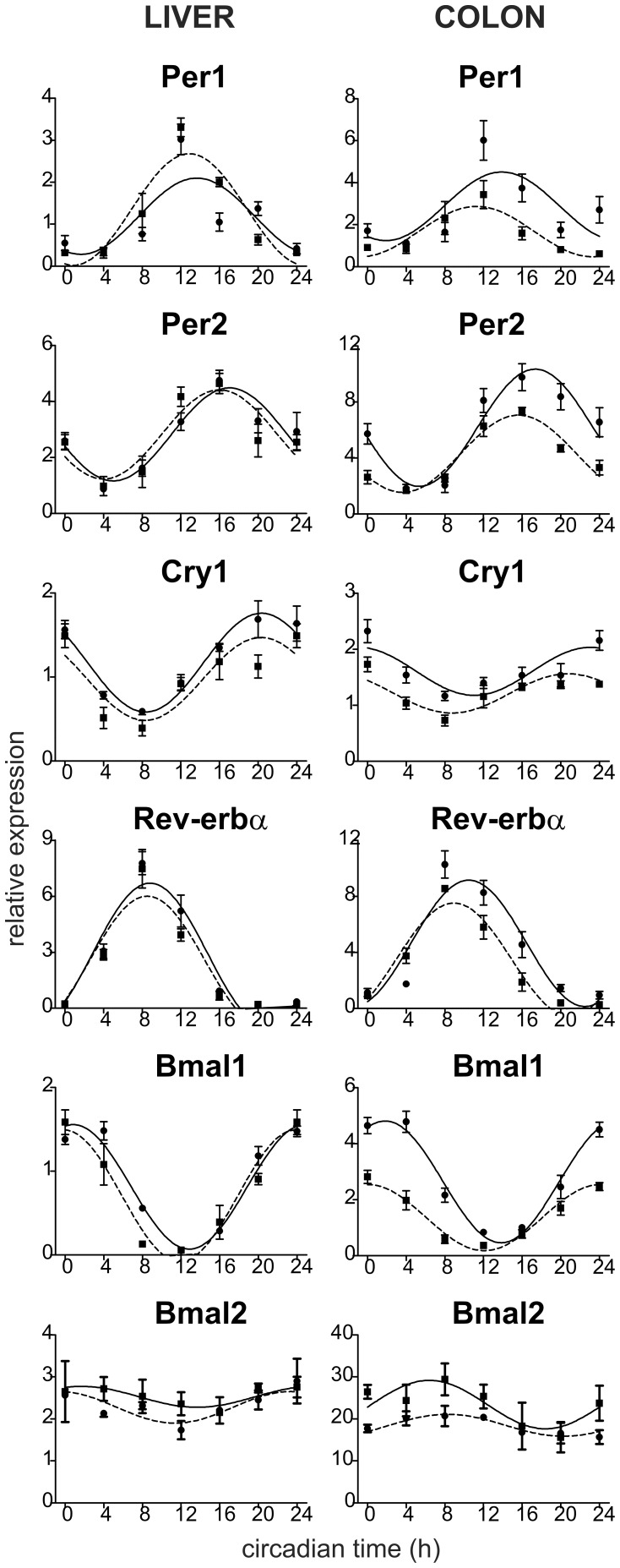
Clock gene expression profiles in the liver (left) and colon (right) of Wistar rats (full line) and SHR (dashed line). The rats were maintained and sampled as described in Fig. 2. Daily profiles of relative expression of *Per1*, *Per2*, *Cry1*, *Rev-erbα*, *Bmal1* and *Bmal2* mRNA levels were determined by RT-PCR. Time is expressed as circadian time (h) with CT0 corresponding to lights-on on the previous LD cycle (for details, see Methods). Data were fitted by cosine curves and each point represents the mean ± S.E.M. of 3 to 5 animals.

**Figure 4:**
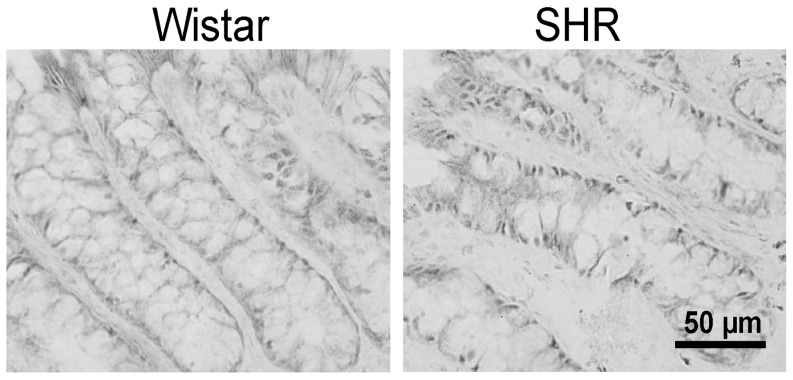
Immunostaining of BMAL1 protein in the colonic epithelium of Wistar rat (left) and SHR (right). Localization of immunopositive cells along the colonic crypt was similar in both rat strains.

Daily profiles in expression of 6 canonical clock genes, namely *Per1*, *Per2*, *Cry1*, *Rev-erbα*, *Bmal1* and *Bmal2*, in the liver and colon were compared between SHR and controls maintained under LD12:12 and sampled on the first cycle in constant darkness (Fig. 3). A cosinor analysis revealed that all of the profiles exhibited circadian rhythms because R^2^ values of the cosine fits were high (Tables S3 and S4). In the liver (Table S3), acrophases of all clock gene expression profiles in SHR did not significantly differ from those in controls. Additionally, the amplitudes and mesors of the profiles of all studied clock genes in the liver of SHR were not different from those of Wistar rats. In the colon of SHR (Table S4), acrophases of *Per2*, *Rev-erbα* and *Bmal1* expression profiles were significantly advanced compared to controls (*Per2*: t = −4.747, DF = 6, P = 0.003; *Rev-erbα*: t = −2.981, DF = 6, P = 0.025; *Bmal1*: t = −6.787, DF = 6, P<0.001), whereas advances in the *Per1*, *Cry1* and *Bmal2* expression profiles were only suggested or not present, respectively. Moreover, amplitudes and/or mesors of all of the clock gene expression rhythms were significantly decreased in SHR compared to controls (*Per1*: mesor t = −3.664, DF = 6, P = 0.011; *Per2*: amplitude t = −3.502, DF = 6, P = 0.013 and mesor t = −6.799, DF = 6, P<0.001; *Cry1*: mesor t = −5.225, DF = 6, P = 0.002; *Rev-erbα*: mesor t = −2.845, DF = 6, P = 0.029; *Bmal1*: amplitude t = −9.161, DF = 6, P<0.001 and mesor: t = −15.880, DF = 6, P<0.001; *Bmal2*: mesor t = −6.945, DF = 6, P<0.001). In the SHR colon, the spatial distribution and number of clock cells, as determined by BMAL1 immunohistochemistry, did not differ from control rats (Fig. 4).

These data demonstrate significant tissue-specific differences between the clock gene expression profiles in SHR and Wistar rats. In the liver, none of the clock gene expression profiles of SHR differed from control rats, whereas in the colon, most of the clock gene expression profiles were phase advanced and the amplitudes and/or mesors of the rhythms were depressed in SHR compared to control rats. Importantly, *Per2*, *Rev-erbα* and *Bmal1* profiles in the colons of SHR were phase-advanced as well as suppressed compared to the profiles in Wistar rats. The suppression did not seem to be due to the reduction in the number of clock cells in the colon of SHR compared with controls.

### Mutual Synchrony between the Phases of the Central and Peripheral Clocks is Changed in SHR Compared to Control Rats

**Figure 5:**
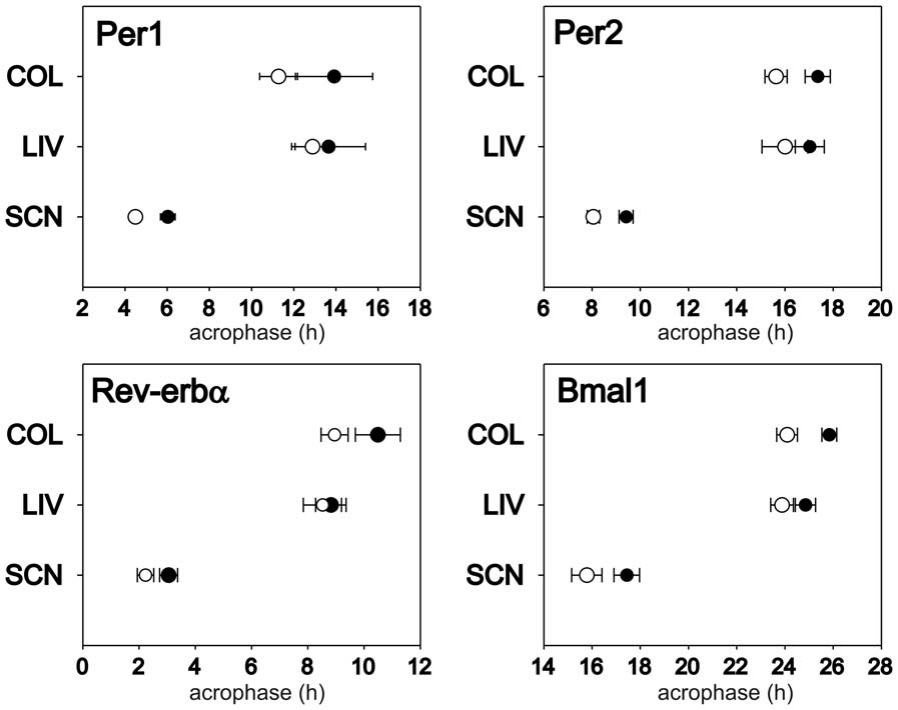
Comparison of acrophases of the circadian rhythms in clock gene expression in the SCN, liver (LIV) and colon (COL). The acrophases in the Wistar rats (full circle) and SHR (open circle) were determined on the basis of cosine fits of *Per1*, *Per2*, *Rev-erbα* and *Bmal1* expression profiles shown in Fig. 2 and 3. Error bars represent S.D.

Comparison of phases between the central clock in the SCN and the peripheral clocks (Fig. 5) revealed that in both strains, the hepatic and colonic clocks are delayed to the SCN by approximately 6–8 h, depending on the gene and strain studied. In controls, *Per1* and *Per2* gene expression profiles in the liver and colon were in the same phase, while *Rev-erbα* and *Bmal1* expression profiles in the liver were slightly phase-advanced relative to those in the colon (t = −3.149, DF = 6, P = 0.020 and t = −3.855, DF = 6, P = 0.008, respectively). In SHR, no significant differences between the phases of the hepatic and colonic clocks were present. Apparently, the hepatic clock was in the same phase in SHR and controls, whereas the SCN and colonic clocks of SHR were phase-advanced compared to controls. Consequently, the mutual phase-relationship between the SCN clock and the peripheral clocks in SHR differed from that observed in controls (Fig. 5).

### Expression Profiles of Clock-controlled and Clock-related Genes Involved in Metabolic Pathways are Affected in the Liver and Colon of SHR Compared to Control Rats

**Figure 6:**
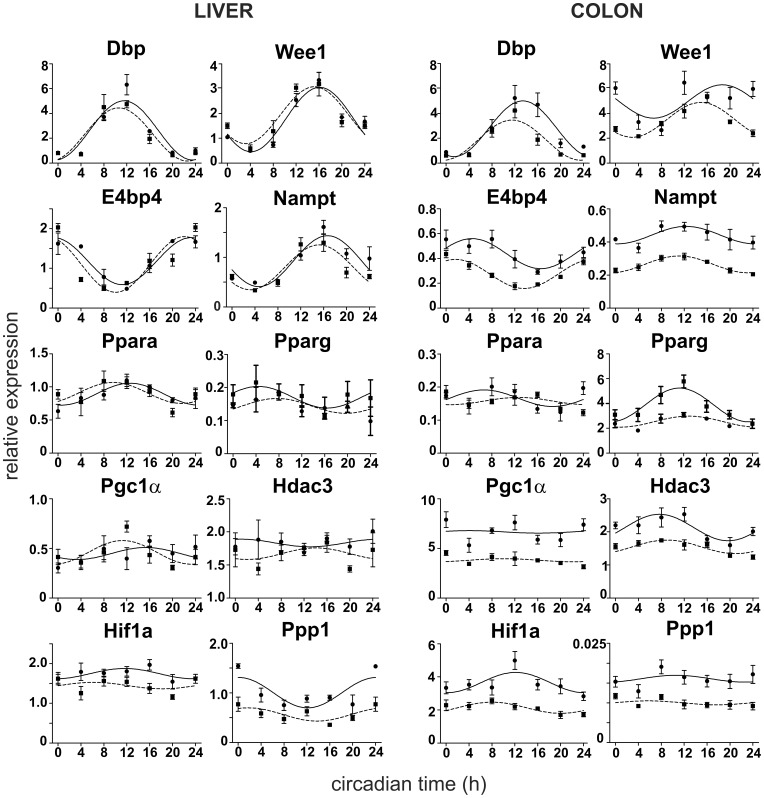
Clock- and metabolism-related gene expression profiles in the liver (left) and colon (right) of Wistar rats (full line) and SHR (dashed line). The rats were maintained and sampled as described in Fig. 2. Daily profiles of relative expression of *Dbp*, *Wee1*, *E4bp4*, *Nampt*, *Ppara*, *Pparg*, *Pgc1α*, *Hdac3*, *Hif1a* and *Ppp1r3c* mRNA levels were determined by RT-PCR. Time is expressed as circadian time (h) with CT0 corresponding to lights-on on the previous LD cycle (for details, see Methods). Data were fitted by cosine curves and each point represents the mean ± S.E.M. of 3 to 5 animals.

The daily expression profiles of 10 clock-controlled and clock-related genes, namely *Dbp*, *Wee1*, *E4bp4*, *Nampt*, *Ppara*, *Pparg*, *Pgc1α*, *Hdac3*, *Hif1a* and *Ppp1r3c*, were determined in the liver and colon of SHR and Wistar rats, which were maintained under LD12:12 and then sampled during the first day in constant darkness (Fig. 6). Results of the cosinor analysis of these profiles, i.e., R^2^ values, acrophases, amplitudes and mesors, are summarized in Tables S3 and S4. In the liver (Table S3), a cosinor analysis revealed significant circadian rhythms in the expression of all studied genes, with the exception of *Hdac3* in both strains, and *Hif1a* in SHR only. In the colon (Table S4), all gene expression profiles exhibited circadian rhythms, with the exception of *Ppp1r3c* and *Pgc1α* in both strains and *Ppara* in SHR only.

*Dbp* expression profile exhibited high amplitude rhythm in the liver, which did not differ between SHR and control rats in acrophase, amplitude or mesor. However, in the colon, the profile in SHR was significantly advanced (t = −3.662, DF = 6, P = 0.011) and depressed (amplitude: t = −2.666, DF = 6, P = 0.037; mesor: t = −5.235, DF = 6, P = 0.002) compared to controls.

*Wee1* expression profile in the liver exhibited high amplitude rhythms in SHR and Wistar rats and there were no differences in acrophase, amplitude or mesor between these two strains. In the colon, the rhythm in SHR was phase-advanced (t = −3.089, DF = 6, P = 0.021) and the mean levels were depressed (t = −4.639, DF = 6, P = 0.004) compared to controls.

*E4bp4* and *Nampt* expression profiles in the liver exhibited high amplitude rhythms in both strains, with no differences in the phase, amplitude or mean values. In SHR and controls, the rhythms exhibited lower amplitudes in the colon compared to the liver. In the colon of SHR, the mean levels were significantly decreased (*E4bp4*: t = −11.697, DF = 6, P<0.001; *Nampt*: t = −23.189, DF = 6, P<0.001) relative to controls. Moreover, *E4bp4* rhythm was significantly phase-advanced in SHR compared to controls (t = −4.784, DF = 6, P = 0.003).

*Ppara* and *Pparg* expression profiles exhibited low amplitude rhythms in the liver of both strains. In the colon, both genes were expressed rhythmically in controls, whereas in SHR, the *Ppara* was expressed constitutively. Moreover, *Pparg* expression profiles in the colon of SHR were significantly suppressed (mesor: t = 10.321, DF = 6, P<0.001; amplitude: t = 5.274, DF = 6, P = 0.002) compared to controls.

*Pgc1α* and *Hdac3* expression in the liver of both strains exhibited only borderline significant rhythms or were expressed constitutively, respectively. Conversely, in the colon *Pgc1α* was expressed constitutively, whereas *Hdac3* exhibited rhythmic expression in both strains. Importantly, the mean expression levels of both genes were significantly lower in the colon of SHR compared to controls (*Pgc1α* mesor: t = −9,668, DF = 6, P<0.001; *Hdac3* mesor: t = −10.815, DF = 6, P<0.001).

*Hif1a* expression profile in the liver exhibited a low amplitude rhythm in controls, but in SHR, this gene was expressed constitutively with decreased mean values (mesor: t = −5.441, DF = 4, P = 0.006) compared to controls. In the colon, the rhythm in *Hif1a* expression was phase-advanced (t = −3.108, DF = 6, P = 0.021), and the mean values were significantly lower (t = −11.443, DF = 6, P<0.001) in SHR relative to controls.

*Ppp1r3c* was expressed rhythmically in the liver and constitutively in the colon of both strains. The mean expression levels in SHR were significantly decreased in the liver (t = −6.083, DF = 4, P = 0.004), as well as in the colon (t = −14.205, DF = 6, P<0.001).

In summary, these data clearly demonstrate that whereas in the liver, the differences in the gene expression profiles between both strains are negligible or marginal, in the colon most of these profiles were significantly different, being phase-advanced (*Dbp*, *Wee1*, *E4bp4*, *Hif1a*) and/or suppressed (all but *Ppara*) in SHR relative to controls.

### Endogenous Period of SHR Fibroblast Clock does not Differ from Wistar Clock

**Figure 7:**
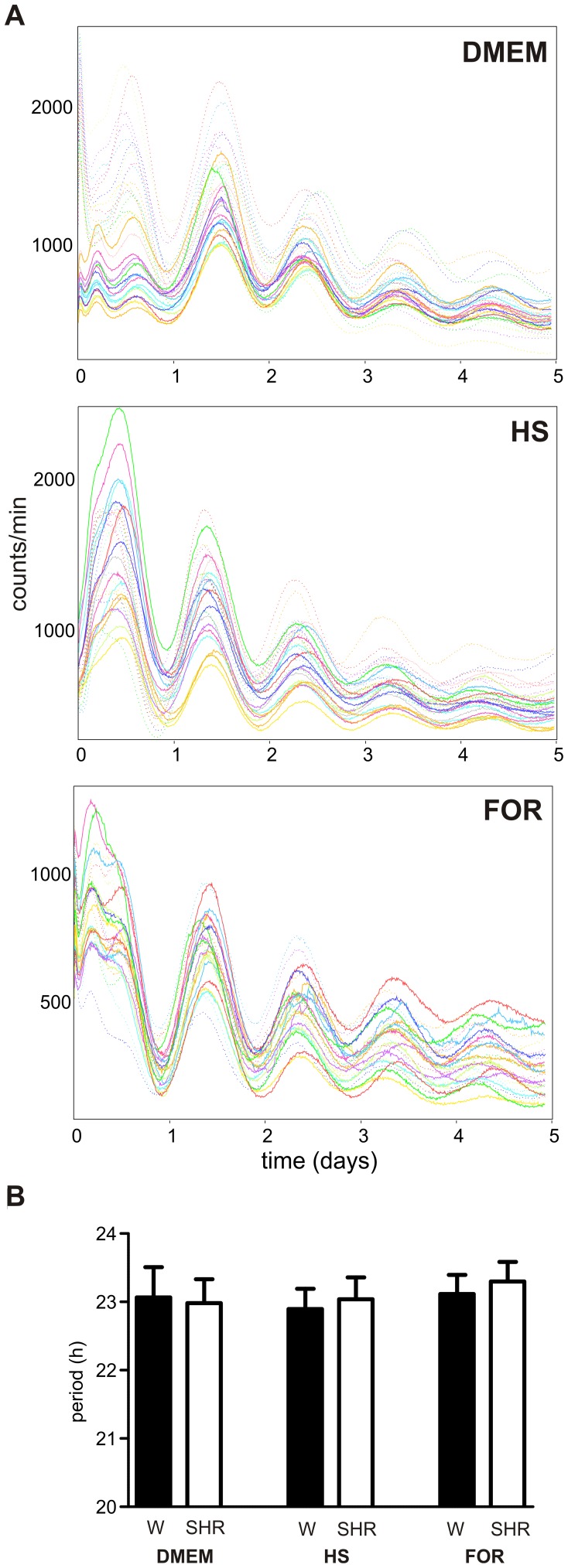
Circadian rhythms of fibroblasts from Wistar rats and SHR. A) Recordings of bioluminescence of spontaneously immortalized fibroblasts from Wistar rats (dotted lines) and SHR (full lines) transfected with Bmal1-dLuc circadian reporter. The fibroblasts were synchronized with simple medium exchange (DMEM), 10 µM Forskolin (FOR) or 50% horse serum (HS). B) Average periods (± S.D.) of the circadian rhythm of spontaneously immortalized fibroblasts from Wistar rats (full column) and SHR (open column) synchronized as described in A. No significant differences between the periods of circadian rhythms in both rat strains were detected.

Because RT PCR analysis of daily profiles in clock gene expression did not allow for the assessment of the endogenous period of the peripheral clock, the circadian period was measured in spontaneously immortalized fibroblasts isolated from SHR and Wistar rats transfected with Bmal1-dLuc circadian reporter. The resulting periods showed no significant differences between either entraining conditions (simple medium exchange, 10 µM Forskolin or 50% horse serum) or strains. The average period was 23.02±0.35 h and 23.08±0.34 h for Wistar and SHR, respectively (Fig. 7). These data suggest that peripheral clocks likely run with the same period in both rat strains.

## Discussion

Although most studies published to date on the circadian aspects of SHR physiology used WKY rats as controls, in our present study we intentionally selected Wistar:Han rats as a control group. This was due to the fact that WKY rats exhibit substantial behavioral, genetic and neurobiological heterogeneity, and the various WKY sub-strains seemed to differ in their suitability as controls for specific experiments (for review, see [37]). For example, some studies suggested that WKY, rather than SHR, show atypical performance in behavioral tests [38], [39]. This heterogeneity is also represented by a high variability of WKY rats in certain circadian parameters among various studies. For example, the circadian period of behavioral activity of WKY rats was either shorter [27] or longer [40] than 24 h. Wistar rats, the original background strain for SHR, represent a well-established rat model that does not have any cardiovascular or metabolic pathology and has also been widely used in chorobiological studies. Therefore, using Wistar rats as a control strain allowed us to correlate the data obtained from SHR using of extensive general knowledge on basic properties of the circadian system in Wistar rats, including their behavioral, neurochemical and metabolic functions.

### The Central Clock in SHR Runs Faster under Constant Conditions and is Advanced under LD Cycle

Behavioral analysis revealed that the free-running period of SHR maintained under constant conditions was significantly shorter than that of Wistar rats. This means that under constant conditions, the SCN clock, which controls the behavioral rhythm, runs faster in SHR relative to control rats. Comparing the circadian profiles of clock gene expressions in the SCN of SHR and control rats revealed significant phase-differences between both strains already on the first day following the release of the rats into constant darkness; the profiles in SHR were phase-advanced compared to those in control rats. However, the phase advance was unlikely simply due to the shorter period in SHR, because the phase advance was much larger (in hours) than would be expected due to the first day of free-running (in minutes). Previous studies, using wheel running rhythm, also reported shorter endogenous period in SHR compared to WKY rats under constant conditions [27], [40]. However, when spontaneous rhythmicity was measured, this effect was [27] or was not [40] present; while in one study releasing of SHR into constant conditions resulted in circadian rhythm free-running with a period much shorter than 24 h [27], in the other study the period was longer than 24 h [40]. It was not clear about what experimental conditions may account for such significant differences. In fact, the activity level may affect circadian period [41] and overall spontaneous and wheel running activity of SHR was found to be higher relative to WKY rats [42]. However, the increase in activity did not seem to be attributed to the shortening of the circadian period [40]. Instead, the behavioral differences between SHR and WKY rats were discussed with respect to a possible higher sensitivity of SHR to stressful experimental environment [43]. In contrast to these previous studies, we were not able to detect any difference in total spontaneous daily activity between SHR and control rats, under either constant or entrained conditions (but see below). Therefore, the shorter period of the free-running activity rhythm is likely not simply due to the overall “hyperactivity” of the SHR.

Under the LD cycle, SHR were more active during the daytime compared to control rats. Especially, they started to be highly active already before the lights-off, which was much earlier than control rats. Analogous to humans, SHR might be considered as a model for early chronotypes due to the positive phase angle of entrainment. Similarly, in a study by Peters et al. [27], SHR also became active much earlier than WKY rats under the LD cycle. As already mentioned, in this study, we found that the advanced onset of activity correlated with advanced profiles of clock gene expression in the SCN in SHR compared to controls. Therefore, it is likely that the different entrainment of the behavioral rhythm in SHR arises from different entrainment of the SCN clock by the LD cycle. Indeed, a difference in light sensitivity of the circadian system to both phase-advancing and phase-delaying light pulses in SHR compared to WKY rats, has been suggested [27]. Therefore, our data support these findings and suggest that in SHR, the SCN might be differently entrained by the LD cycle. Alternatively, we also cannot exclude a possibility that the increased activity during the daytime is due to a weaker amplitude of the overt rhythm (as discussed below). If so, it is plausible to speculate that increased daytime activity might feed-back on the SCN, thereby resulting in a phase advance of the clock. Such feedback mechanism has previously been suggested in other animal models [44].

### The Amplitude of Behavioral Output Rhythm is Dampened in SHR

As previously discussed, SHR maintained under LD cycle had significantly lower ratio of activity during the nighttime and inactivity during the daytime compared to control rats. Similarly, feeding, which is another output rhythm driven by the SCN, also exhibited a decrease of amplitude in SHR maintained under the LD cycle relative to WKY rats [45]. However, in our study, the rhythm in locomotor activity exhibited a lower amplitude in SHR compared to control rats not only under entrained but also under free-running conditions. Therefore, it is unlikely that the decrease in the amplitude is simply due to a weak entrainment of the rhythm by the LD cycle. Instead, the data favor the hypothesis that it might be due to a lower amplitude of the rhythmical output signal generated spontaneously by the SCN clock in SHR. The mechanism of the amplitude decline does not seem to reside in the molecular clock itself because the amplitudes of the clock gene expression profiles in the SCN did not differ between SHR and control rats. Therefore, the oscillatory capacity of the SCN clock is the same in both rat strains, but its capability to drive the output rhythms is likely deteriorated in SHR.

### Peripheral Clock Gene Expression and Mutual Phasing among the Central SCN Clock and Peripheral Clocks are Altered in SHR

Clock gene expression profiles in the hepatic and colonic peripheral clocks exhibited significant tissue-specific differences between SHR and Wistar rats. The phases and amplitudes of the hepatic clocks did not differ between both strains, whereas the colonic clocks were phase advanced and suppressed in SHR compared to controls. Similarly, Cui *et al.* also did not find any differences in clock gene expression profiles between SHR and WKY rats in the liver [45]. Importantly, the suppression of the rhythms in the colon was likely not due to differences in the spatial distribution of colonic cells containing circadian clock between SHR and controls, which was demonstrated by detection of BMAL1-immunopositive cells. In the colon, specifically *Per2*, *Rev-erbα* and *Bmal1* profiles of SHR were phase advanced, as well as suppressed, compared with the profiles of Wistar rats. These genes represent canonical clock components that receive rhythmical signals mostly from the SCN (*Per2*) or are solely part of the peripheral clockwork (*Rev-erbα* and *Bmal1*) [46]. Thus, reduced SCN signaling as well as suppressed rhythmicity of the peripheral colonic clock was suggested in SHR. The dampened amplitude of the colonic clock in SHR is in accordance with the above mentioned hypothesis of a weaker signaling from the central clock to the periphery. However, this effect seems to be tissue-specific because, in contrast to the colon, the hepatic clock of SHR was not suppressed. The tissue-specificity excludes the possibility that the advance of the peripheral clocks was due to the advance in the SCN-driven feeding behavior. Similar to our data, tissue-specific differences in clock gene expression were detected in a study by Cui et al., which demonstrated no differences in clock gene expression profiles in liver but suppression of the *Rev-erbα* and advance of *Bmal1* expression profiles in the heart of SHR compared to WKY rats [45]. Our data show that in SHR the liver clock was not advanced to the same extent as that in the SCN. This might suggest involvement of different pathways mediating the SCN signal to the liver and colon. Consequently, the mutual phase-relationship between the central clock in the SCN and the peripheral clocks in the liver and colon, and possibly the heart, seems to differ in SHR compared to Wistar rats.

### Period of the Peripheral Clock in SHR and Wistar Rats does not Differ

Based on the differences in circadian periods of behavioral activity rhythms in SHR and controls, the central SCN clock, which drives the rhythmicity, seemed to run faster in SHR compared to Wistar rats (see above). In contrast, no differences in periods or amplitudes between both strains were detected during various entraining conditions in fibroblasts, thereby suggesting that the in vitro SHR molecular core clockwork in periphery was not compromised. Therefore, our data strongly suggest that the polymorphisms in the SHR *Bmal1* promoter [30] likely do not influence the core molecular clockwork in the fibroblasts. Moreover, the outcome of our study clarifies questions related to the possibility that the circadian phenotype of SHR is caused by a mutation in the clock gene coding sequence. This possibility has been suggested based on the published SHR genome sequence, which showed frameshift insertions in the *Bmal2* gene [47]. However, recently we demonstrated that the full-length cDNA sequences of the *Bmal2* gene in both SHR and Wistar rats were not different (GenBank JQ361086).

### The Clock- and Metabolism-related Gene Expression Profiles in SHR are Advanced and/or Suppressed in the Colon, but not in the Liver

In order to ascertain the functional properties of the circadian clock in the liver and colon of SHR and control rats, we selected several clock- and metabolism-related genes and analyzed their daily expression profiles. The selected genes were i) clock-controlled (*Dbp, E4bp4*) and clock-related (*Hif1a*) transcription factors that regulate various metabolic pathways, ii) nuclear receptors (*Ppara, Pparg*) and their co-activators (*Pgc1α*) that regulate metabolism, and iii) enzymes involved in posttranslational modification (*Wee1, Ppp1r3c, Hdac3*) and NAD^+^ biosynthesis (*Nampt*). In the liver, there were either negligible or only marginal differences detected in the gene expression profiles between SHR and controls, whereas these differences were profound in the colon. Most of the studied profiles differed significantly, and were phase-advanced (*Dbp*, *Wee1*, *E4bp4*, *Hif1a*) and/or suppressed (all but *Ppara*) in the colon of SHR compared to controls. These results are in accordance with our finding that in the liver the molecular clock mechanism of SHR did not differ from controls, whereas in the colon of SHR, the clock was advanced and/or suppressed. Thus, a relationship between the anomalies in the temporal control of circadian and metabolic transcriptome is highly suggested. However, it is difficult to determine the cause and effect relationship because an impaired SHR clock may directly and indirectly influence metabolism and, at the same time, systemic changes in SHR metabolism may feedback on the clock mechanism.

The mechanism of how circadian regulation of gene expression in the colon is distorted likely involves a weakening of E-box-driven transcription, because rhythms of genes which are activated by BMAL1:CLOCK via this pathway, namely *Dbp*, *Wee1*, *Nampt, Ppara, Pparg* and *Pgc1α* [48], [49], [50], [51], [52], [53], were suppressed. The weakening might arise from reduced levels of *Bmal1* transcription in the colon of SHR. *Dbp* is a clock-controlled gene whose protein product binds to D-elements on promoters of other genes and may thus temporally control a relatively large portion of the rhythmic cellular transcriptome that is related to metabolism [54], [55], [56]. Therefore, due to this suppression, the temporal control of the metabolic processes may be less pronounced in the colon of SHR compared to controls. Moreover, regulation of transcription via binding of REV-ERBα to ROR elements on promoters of some genes, including *E4bp4* [57], which occurs in opposite phase to the E-box-mediated transcription, seemed also to be decreased, because the rhythm of *E4bp4* was suppressed. *E4bp4* is a bZIP factor which acts as an active transcriptional repressor on E4BP4-binding sites on promoters of other genes, including *Per2,* to repress their transcription [58], [59], [60]. Therefore, our data demonstrate that both arms of the clock, which serve to transduce rhythmical signal in anti-phase out of the clock, are compromised in the colon of SHR. Due to the impairment of the clock output mechanisms, expression of various genes involved in pathways important for cell physiology is likely affected in the colon of SHR. For example, the severe suppression of expression of clock-driven gene *Nampt*, i.e., the gene encoding rate-limiting enzyme involved in nicotinamide adenine dinucleotide (NAD^+^) biosynthesis [53], [61], may likely result in lower levels of NAD^+^ in the colonic cells. Nuclear receptors *Ppara* and *Pparg* are transcription factors which are widely expressed and have central roles in adipogenesis, lipid and glucose homeostasis, but are also implicated in linking circadian clock and metabolism [62], [63]. Notably, the rhythm in expression of *Ppara* was abolished and that of *Pparg* was severely suppressed in colon of SHR. Similarly, the *Ppara* expression in the heart, but not in liver, differed between SHR and WKY rats [45]. The reduced expression of *Pgc1α*, a co-activator enhancing activity of many nuclear receptors, which include PPARs, might also result in serious changes in metabolic state of the colonocytes. The PGC1α-dependent metabolic transcription is activated by pathways that sense the energy status in a cell, such as the AMP/ATP ratio via the phosphorylation by AMPK, and the NAD+/NADH ratio via deacetylation by Sirtuin 1 [64]. Importantly, both AMPK and Sirtuin1 are intimately communicating with the core clock mechanism [65], [66], [67]. PGC1α is also directly linked to molecular clock mechanism as it serves as co-activator of RORγ, an orphan nuclear receptor that activates *Bmal1* transcription [68]. Suppressed expression of *Pgc1α* might thus contribute to the suppression of the *Bmal1* expression rhythm in the colon of SHR. Because all of these affected genes are sensitive to the metabolic state of the cell, the changes of clock mechanism we observed in the colon of SHR might also arise from a weakening in the feedback that the cellular metabolism imposes on the peripheral clock.

The suppressed expression of *Wee1, Hdac3*, *Hif1a* and *Ppp1r3c* demonstrated the broad impairment in the cellular physiology of the colon in SHR compared to controls. *Wee1* kinase is a negative regulator of mitotic entry, and as a clock-controlled gene, it temporally controls cell cycle [52]. The mechanism is particularly relevant to the colon, where epithelial cells undergo cell division and differentiation to enterocytes, and where *Wee1* is expressed rhythmically [69]. *Hdac3* encodes a protein that belongs to the histone deacetylase family and participates in regulation of metabolism, cell growth and apoptosis [70], [71], [72], [73]. HDAC3 influences the expression of a large number of genes, including *Pparg* [74] and *Hif1a* [75]. *Hif1a* encodes the alpha subunit of hypoxia-inducible factor-1 (HIF1), a bHLH-PAS transcription factor, which is a master metabolic and apoptotic regulator that controls homeostatic responses to hypoxia (reviewed by [76]). It regulates genes containing HIF-responsive elements (HREs), which includes *Ppp1r3c,* a phosphoprotein phosphatase 1 regulatory subunit 3c [77]. *Ppp1r3c* is one of the many subunits of PP1, a major regulator of metabolism, cell cycle, apoptosis and also the circadian clock [78], [79], [80].

Surprisingly, all these aberrant expression profiles were present selectively in the colon but not in the liver. Similarly, a study that compared the gene expression profiles in the liver and heart of SHR and WKY rats also resulted in conclusion that the clock gene expression was more severely impaired in the heart than in the liver [45]. These findings may indicate that the hepatic clock is more autonomous and thus less sensitive to SCN signaling than the clocks in the colon and heart. Indeed, the rhythmicity of clock gene expression in animals with distorted SCN signaling, via prolonged exposure to constant light, was better rescued by regular feeding regime in the liver than in the colon [81]. Nevertheless, the unaffected hepatic clock in this experiment served us as a control to provide evidence that the suppressed clock and clock-controlled gene expression we observed were not simply due to some tissue-unspecific genetic differences between the SHR and Wistar strains.

### Conclusion

Our data demonstrated significant alterations of the circadian system in SHR compared to Wistar rats. The observed changes may result from various allelic alterations in the SHR genome (Atanur et al., 2010) that may have no effect on hypertension or metabolism. Nevertheless, our data allow us to speculate about a causal relationship between the altered circadian system and pathophysiology of SHR. The central SCN clock in SHR appears to be differently entrained with the LD cycle and at the same time and it is likely not able to distribute robust circadian signal necessary to drive output rhythms as compared to Wistar rats. The malfunction of the circadian SCN clock leads to impairment of internal synchrony within the circadian system. This internal desynchrony may contribute to a weakening in the temporal control of gene expression in the colon, thus affecting its ability to adjust properly to daily changes in the external environment. At the same time, the impaired feedback from metabolism might also contribute to weaker clock oscillation in the SHR colonocytes. Altogether, our data revealed that SHR exhibit an early chronotype-like behavior and demonstrate tissue-specific malfunction of the circadian and metabolic pathways in the colon of SHR.

## Supporting Information

Table S1 **List of genes analyzed by Q RT-PCR with sequences of used primers.**
(DOC)

Table S2 **Cosinor analysis of SCN expression profiles.**
(DOC)

Table S3 **Cosinor analysis of liver expression profiles.**
(DOC)

Table S4 **Cosinor analysis of colon expression profiles.**
(DOC)
