# Supplementary material for: Early Chronotype and Tissue-Specific Alterations of Circadian Clock Function in Spontaneously Hypertensive Rats
Source: PLoS One. 2012 Oct 2;7(10):e46951. doi: 10.1371/journal.pone.0046951 (PMC3462770; doi:10.1371/journal.pone.0046951)
Supplement: Table S1 — List of genes analyzed by Q RT-PCR with sequences of used primers. (DOC) [file pone.0046951.s001.doc]

Table S1. Primer sequences.

| **Gene** | **GenBank no.** | **Forward** | **Reverse** | **source** |
| --- | --- | --- | --- | --- |
| B2m | NM_012512 | CGCTCGGTGACCGTGATCTTTCTG | CTGAGGTGGGTGGAACTGAGACACG | M.S. |
| Per1 | AB002108 | CGCACTTCGGGAGCTCAAACTTC | GTCCATGGCACAGGGCTCACC | M.S. |
| Per2 | NM_031678 | CACGCAACGGGGAGTACATCACAC | CAAGGGGAGGCTGCGAACACAT | M.S. |
| Rev-erbα | NM_145775 | GCTGTGCGGGAGGTGGTAGAAT | TGTAGGTTGTGCGGCTCAGGAA | M.S. |
| Cry1 | NM_198750 | GTGGTGGCGGAAACTGCTCTC | ACTCTGTGCGTCCTCTTCCTGA | M.S. |
| Bmal1 | AB012600 | CAATGCGATGTCCCGGAAGTTAGA | TCCCTCGGTCACATCCCTGAGAAT | M.S. |
| Bmal2 | XM_001075231 | GCCGGGCATATCCACAGG | GGCGCTCCCGAAGTTTACAC | M.S. |
| Dbp | NM_012543 | TTTGCGCCGCTGCTGTGGGAACG | GGGGGAGGGCGCGGGAGTGC | M.S. |
| Wee1 | NM_001012742 | ATCCCCATGTGGTTCGTTATTTCTCTG | ATGTATGTATCTCAAGCCCCTGCCAACT | M.S. |
| E4bp4 | NM_053727 | GCAGGAGCCCGTGGAGTTGGAGAG | AGGAGGGGAGGGGAGTGGGAGTAGGT | M.S. |
| Nampt | NM_177928 | CTTTGGTTCTGGTGGCGCTTTGCTAC | GCCGGCCCTTTTTCGACCTTTTGTT | M.S. |
| Ppara | NM_013196 | ACTATGGAGTCCACGCATGTGA | TTGTCGTACGCCAGCTTTAGC | Db |
| Pparg | NM_013124 | CACAATGCCATCAGGTTTGG | GCTGGTCGATATCACTGGAGATC | Db |
| Pgc1α | NM_031347 | CCGTAAATCTGCGGGATGATG | CAGTTTCATTCGACCTGCGTAA | M.S. |
| Hdac3 | NM_053448 | ACCCCAGCCCCTCTTCCCCTTATTTG | GGTTGGCATGCCTTCCTCCCCTCAG | M.S. |
| Hif1a | NM_024359 | AGCGGCTGGGGACACGAT | TGGCTTTGGAGTTTCAGAGGCAGGTAA | M.S. |
| Pp1r3c | NW_047565 | CCGCTAAGTGCGTGGTGCGA | GGGGTGGTGAATGTGCCAAGCA | M.S. |

List of genes analyzed by Q RT-PCR with sequences of used primers. M.S. (designed by M. Sladek); Db (<http://www.rtprimerdb.org/>).
