# Supplementary material for: Early Chronotype and Tissue-Specific Alterations of Circadian Clock Function in Spontaneously Hypertensive Rats
Source: PLoS One. 2012 Oct 2;7(10):e46951. doi: 10.1371/journal.pone.0046951 (PMC3462770; doi:10.1371/journal.pone.0046951)
Supplement: Table S2 — Cosinor analysis of SCN expression profiles. (DOC) [file pone.0046951.s002.doc]

Table S2. Cosinor analysis of SCN expression profiles.

|  |  | **Per1** | | **Per2** | | **Rev-erbα** | | **Bmal1** | |
| --- | --- | --- | --- | --- | --- | --- | --- | --- | --- |
|  |  | **Wistar** | **SHR** | **Wistar** | **SHR** | **Wistar** | **SHR** | **Wistar** | **SHR** |
| **Acrophase** | | 6.030 | 4.487 | 9.413 | 8.052 | 3.045 | 2.232 | 17.440 | 15.779 |
| **SD** | | 0.354 | 0.222 | 0.290 | 0.250 | 0.317 | 0.294 | 0.534 | 0.626 |
| **Amplitude** | | 0.102 | 0.100 | 0.095 | 0.119 | 0.088 | 0.098 | 0.045 | 0.043 |
| **SD** | | 0.011 | 0.006 | 0.007 | 0.008 | 0.007 | 0.007 | 0.007 | 0.008 |
| **Mesor** | | 0.140 | 0.130 | 0.152 | 0.155 | 0.184 | 0.170 | 0.115 | 0.104 |
| **SD** | | 0.007 | 0.004 | 0.005 | 0.006 | 0.005 | 0.005 | 0.005 | 0.005 |
| **R^2^** | | 0.835 | 0.927 | 0.912 | 0.915 | 0.891 | 0.911 | 0.691 | 0.639 |

R^2^ (coefficient of determination)
