# Supplementary material for: Early Chronotype and Tissue-Specific Alterations of Circadian Clock Function in Spontaneously Hypertensive Rats
Source: PLoS One. 2012 Oct 2;7(10):e46951. doi: 10.1371/journal.pone.0046951 (PMC3462770; doi:10.1371/journal.pone.0046951)
Supplement: Table S3 — Cosinor analysis of liver expression profiles. (DOC) [file pone.0046951.s003.doc]

Table S3. Cosinor analysis of liver expression profiles.

| **Liver** | **Per1** | | **Per2** | | **Cry1** | | **Rev-erbα** | | **Bmal1** | | **Bmal2** | | **Dbp** | | **Wee1** | |
| --- | --- | --- | --- | --- | --- | --- | --- | --- | --- | --- | --- | --- | --- | --- | --- | --- |
|  | **Wistar** | **SHR** | **Wistar** | **SHR** | **Wistar** | **SHR** | **Wistar** | **SHR** | **Wistar** | **SHR** | **Wistar** | **SHR** | **Wistar** | **SHR** | **Wistar** | **SHR** |
| **Acro** | 13.653 | 12.887 | 17.027 | 16.007 | 20.359 | 20.307 | 8.823 | 8.538 | 24.852 | 23.880 | 25.668 | 23.370 | 11.510 | 10.618 | 16.122 | 15.205 |
| **SD** | 1.753 | 0.826 | 0.597 | 0.953 | 0.422 | 1.221 | 0.543 | 0.683 | 0.422 | 0.468 | 1.115 | 3.692 | 1.004 | 0.886 | 0.723 | 0.856 |
| **Amp** | 0.908 | 1.330 | 1.662 | 1.594 | 0.588 | 0.493 | 3.813 | 3.470 | 0.743 | 0.786 | 0.248 | 0.376 | 2.380 | 2.142 | 1.296 | 1.146 |
| **SD** | 0.384 | 0.257 | 0.290 | 0.424 | 0.068 | 0.165 | 0.548 | 0.640 | 0.073 | 0.085 | 0.037 | 0.157 | 0.554 | 0.452 | 0.263 | 0.260 |
| **Mesor** | 1.191 | 1.350 | 2.826 | 2.824 | 1.172 | 0.978 | 2.890 | 2.531 | 0.813 | 0.707 | 2.526 | 2.274 | 2.632 | 2.304 | 1.756 | 1.931 |
| **SD** | 0.283 | 0.193 | 0.195 | 0.291 | 0.047 | 0.114 | 0.385 | 0.446 | 0.055 | 0.064 | 0.054 | 0.099 | 0.418 | 0.336 | 0.180 | 0.183 |
| **R^2^** | 0.585 | 0.871 | 0.892 | 0.782 | 0.950 | 0.694 | 0.925 | 0.882 | 0.963 | 0.955 | 0.742 | 0.673 | 0.822 | 0.850 | 0.860 | 0.831 |

| **Liver** | **E4bp4** | | **Nampt** | | **Ppara** | | **Pparg** | | **Pgc1α** | | **Hdac3** | | **Hif1a** | | **Ppp1r3c** | |
| --- | --- | --- | --- | --- | --- | --- | --- | --- | --- | --- | --- | --- | --- | --- | --- | --- |
|  | **Wistar** | **SHR** | **Wistar** | **SHR** | **Wistar** | **SHR** | **Wistar** | **SHR** | **Wistar** | **SHR** | **Wistar** | **SHR** | **Wistar** | **SHR** | **Wistar** | **SHR** |
| **Acro** | 23.223 | 22.070 | 16.674 | 15.207 | 12.585 | 9.601 | 4.420 | 7.662 | 15.331 | 11.282 | - | - | 11.640 | - | 0.128 | 1.781 |
| **SD** | 0.565 | 1.211 | 0.772 | 0.878 | 1.136 | 1.809 | 1.425 | 2.435 | 3.435 | 2.071 | - | - | 2.148 | - | 2.109 | 2.284 |
| **Amp** | 0.592 | 0.701 | 0.513 | 0.452 | 0.165 | 0.158 | 0.033 | 0.022 | 0.060 | 0.119 | - | - | 0.129 | - | 0.306 | 0.133 |
| **SD** | 0.078 | 0.208 | 0.114 | 0.105 | 0.044 | 0.072 | 0.013 | 0.015 | 0.055 | 0.057 | - | - | 0.064 | - | 0.149 | 0.074 |
| **Mesor** | 1.182 | 1.098 | 0.922 | 0.798 | 0.890 | 0.909 | 0.171 | 0.145 | 0.448 | 0.460 | 1.832 | 1.671 | 1.748 | 1.447 | 1.004 | 0.566 |
| **SD** | 0.059 | 0.152 | 0.077 | 0.074 | 0.033 | 0.052 | 0.009 | 0.011 | 0.039 | 0.043 | 0.045 | 0.067 | 0.049 | 0.083 | 0.113 | 0.054 |
| **R^2^** | 0.935 | 0.742 | 0.836 | 0.824 | 0.783 | 0.550 | 0.605 | 0.346 | 0.231 | 0.519 | 0.189 | 0.184 | 0.503 | 0.097 | 0.513 | 0.451 |

Acro (acrophase); Amp (amplitude); R^2^ (coefficient of determination).
