# Supplementary material for: Early Chronotype and Tissue-Specific Alterations of Circadian Clock Function in Spontaneously Hypertensive Rats
Source: PLoS One. 2012 Oct 2;7(10):e46951. doi: 10.1371/journal.pone.0046951 (PMC3462770; doi:10.1371/journal.pone.0046951)
Supplement: Table S4 — Cosinor analysis of colon expression profiles. (DOC) [file pone.0046951.s004.doc]

Table S4. Cosinor analysis of colon expression profiles.

| **Colon** | **Per1** | | **Per2** | | **Cry1** | | **Rev-erbα** | | **Bmal1** | | **Bmal2** | | **Dbp** | | **Wee1** | |
| --- | --- | --- | --- | --- | --- | --- | --- | --- | --- | --- | --- | --- | --- | --- | --- | --- |
|  | **Wistar** | **SHR** | **Wistar** | **SHR** | **Wistar** | **SHR** | **Wistar** | **SHR** | **Wistar** | **SHR** | **Wistar** | **SHR** | **Wistar** | **SHR** | **Wistar** | **SHR** |
| **Acro** | 13.910 | 11.281 | 17.359 | 15.633 | 23.055 | 20.732 | 10.489 | 8.946 | 1.836 | 0.087 | 6.396 | 8.275 | 13.387 | 11.516 | 18.631 | 14.901 |
| **SD** | 1.833 | 0.898 | 0.517 | 0.458 | 1.499 | 1.232 | 0.800 | 0.476 | 0.307 | 0.430 | 1.065 | 0.820 | 0.598 | 0.868 | 1.974 | 0.640 |
| **Amp** | 1.632 | 1.196 | 4.186 | 2.761 | 0.428 | 0.350 | 4.511 | 4.041 | 2.176 | 1.178 | 5.767 | 2.609 | 2.243 | 1.617 | 1.341 | 1.403 |
| **SD** | 0.732 | 0.250 | 0.638 | 0.345 | 0.151 | 0.115 | 0.865 | 0.506 | 0.163 | 0.117 | 3.337 | 1.347 | 0.320 | 0.325 | 0.781 | 0.233 |
| **Mesor** | 2.877 | 1.670 | 6.155 | 4.296 | 1.605 | 1.211 | 4.644 | 3.476 | 2.634 | 1.356 | 23.410 | 18.470 | 2.767 | 1.849 | 4.967 | 3.487 |
| **SD** | 0.536 | 0.188 | 0.426 | 0.239 | 0.113 | 0.081 | 0.641 | 0.357 | 0.120 | 0.088 | 1.163 | 0.376 | 0.238 | 0.245 | 0.522 | 0.166 |
| **R^2^** | 0.557 | 0.851 | 0.915 | 0.942 | 0.670 | 0.702 | 0.873 | 0.942 | 0.978 | 0.962 | 0.733 | 0.854 | 0.925 | 0.861 | 0.425 | 0.902 |

| **Colon** | **E4bp4** | | **Nampt** | | **Ppara** | | **Pparg** | | **Pgc1α** | | **Hdac3** | | **Hif1a** | | **Ppp1r3c** | |
| --- | --- | --- | --- | --- | --- | --- | --- | --- | --- | --- | --- | --- | --- | --- | --- | --- |
|  | **Wistar** | **SHR** | **Wistar** | **SHR** | **Wistar** | **SHR** | **Wistar** | **SHR** | **Wistar** | **SHR** | **Wistar** | **SHR** | **Wistar** | **SHR** | **Wistar** | **SHR** |
| **Acro** | 4.526 | 1.611 | 12.304 | 11.088 | 6.623 | - | 11.252 | 12.889 | - | - | 7.650 | 8.946 | 12.136 | 8.017 | - | - |
| **SD** | 0.912 | 0.653 | 1.369 | 0.361 | 2.501 | - | 0.848 | 1.526 | - | - | 1.057 | 1.462 | 2.009 | 1.344 | - | - |
| **Amp** | 0.121 | 0.117 | 0.053 | 0.051 | 0.025 | - | 1.358 | 0.442 | - | - | 0.399 | 0.204 | 0.613 | 0.337 | - | - |
| **SD** | 0.032 | 0.018 | 0.017 | 0.004 | 0.018 | - | 0.269 | 0.158 | - | - | 0.120 | 0.078 | 0.284 | 0.126 | - | - |
| **Mesor** | 0.439 | 0.275 | 0.441 | 0.264 | 0.166 | 0.158 | 3.882 | 2.537 | 6.666 | 3.811 | 2.131 | 1.548 | 3.659 | 2.135 | 0.016 | 0.010 |
| **SD** | 0.021 | 0.014 | 0.013 | 0.003 | 0.012 | 0.010 | 0.202 | 0.118 | 0.472 | 0.212 | 0.081 | 0.055 | 0.215 | 0.087 | 0.001 | 0.000 |
| **R^2^** | 0.787 | 0.911 | 0.714 | 0.972 | 0.315 | 0.145 | 0.865 | 0.663 | 0.008 | 0.067 | 0.737 | 0.633 | 0.537 | 0.644 | 0.127 | 0.104 |

Acro (acrophase); Amp (amplitude); R^2^ (coefficient of determination).
